# Emerging Role of Neuropilin-1 and Angiotensin-Converting Enzyme-2 in Renal Carcinoma-Associated COVID-19 Pathogenesis

**DOI:** 10.3390/idr13040081

**Published:** 2021-10-16

**Authors:** Md. Golzar Hossain, Sharmin Akter, Md Jamal Uddin

**Affiliations:** 1 Department of Microbiology and Hygiene, Bangladesh Agricultural University, Mymensingh 2202, Bangladesh; 2 Department of Physiology, Bangladesh Agricultural University, Mymensingh 2202, Bangladesh; sharmin.akter@bau.edu.bd; 3 Graduate School of Pharmaceutical Sciences, College of Pharmacy, Ewha Womans University, Seoul 03760, Korea; 4 ABEx Bio-Research Center, East Azampur, Dhaka 1230, Bangladesh

**Keywords:** NRP1, ACE2, COVID-19, renal carcinoma, KIRC, KIRP

## Abstract

Neuropilin-1 (NRP1) is a recently identified glycoprotein that is an important host factor for SARS-CoV-2 infection. On the other hand, angiotensin-converting enzyme-2 (ACE2) acts as a receptor for SARS-CoV-2. Additionally, both NRP1 and ACE2 express in the kidney and are associated with various renal diseases, including renal carcinoma. Therefore, the expression profiles of NRP1 and ACE2 in kidney renal clear cell carcinoma (KIRC) and kidney renal papillary cell carcinoma (KIRP) patients from the various cancer databases were investigated along with their impact on patients’ survivability. In addition, coexpression analysis of genes involved in COVID-19, KIRC, and KIRP concerning NRP1 and ACE2 was performed. The results demonstrated that both t NRP1 and ACE2 expressions are upregulated in KIRC and KIRP compared to healthy conditions and are significantly correlated with the survivability rate of KIRC patients. A total of 128 COVID-19-associated genes are coexpressed, which are positively associated with NRP1 and ACE2 both in KIRC and KIRP. Therefore, it might be suggested that, along with the ACE2, high expression of the newly identified host factor NRP1 in renal carcinomas may play a vital role in the increased risk of SARS-CoV-2 infection and survivability of COVID-19 patients suffering from kidney cancers. The findings of this investigation will be helpful for further molecular studies and prevention and/or treatment strategies for COVID-19 patients associated with renal carcinomas.

## 1. Introduction

Neuropilin-1 (NRP1) is a multifaceted transmembrane glycoprotein that acts as a receptor for semaphorins (SEMA3A) and vascular endothelial growth factor (VEGF) family members [1,2]. Angiogenesis is believed to be influenced by NRP1 binding with VEGF-A, VEGF-B, VEGF-E, PlGF, and HGF/SF [3]. In addition, NRP1 also acts as a pro-angiogenic co-receptor by binding with the hepatocyte growth factor (HGF) in endothelial cells and enhancing the pro-angiogenic cytokines, VEGF-A, and HGF [3]. Moreover, VEGF plays a vital role in angiogenesis and generates tumor growth, tissue repair, and blood vessel networks. Therefore, VEGF and its receptors (VEGFR1 and VEGFR2, and NRP1) are targeted for treating cancer and vascular diseases [1]. Angiotensin-converting enzyme-2 (ACE2) is reported to be highly expressed in the proximal tubular cells of the kidney and regulates renal disease progression [4,5].

ACE2 is reported to be a receptor for SARS-CoV-2 in kidney injury, while, recently, NRP1 has been identified as a potential host factor for SARS-CoV-2, and NRP1 specifically binds with the C-end rule (CendR) motif of the S1 subunit of the spike protein [6,7]. Moreover, inhibiting this binding by monoclonal antibodies, small-molecule inhibitors, or RNA interference significantly reduced the SARS-CoV-2 entry in the cell culture, suggesting a potential therapeutic target for COVID-19 [6].

On the other hand, along with the lung, multiple organs such as the liver, kidneys, heart, and brain might be affected by SARS-CoV-2 in COVID-19 patients [8,9]. SARS-CoV-2 might cause acute kidney injury leading to increased hospital death of COVID-19 patients [10,11,12]. Importantly, a study revealed an increased incidence of SARS-CoV-2 infection in renal cell carcinoma patients with cancer progression and a higher mortality rate [13].

Therefore, in this study, we investigated the expression profile of NRP1 and ACE2 in kidney renal clear cell carcinoma (KIRC) and kidney renal papillary cell carcinoma (KIRP) patients from various cancer databases. In addition, the relationship of NRP1 and ACE2 expression to patients’ survivability rate followed by the coexpression analysis of genes positively regulated with these factors and associated with COVID-19 were studied.

## 2. Materials and Methods

Gene Expression Profiling Interactive Analysis 2 (GEPIA2) server (http://gepia2.cancer-pku.cn/#index), a publicly accessible online database, was used to analyze the NRP1 expression profile across thirty-three (33) human cancers and paired normal tissues using the TCGA (The Cancer Genome Atlas) datasets [14]. Expression data of RNA sequencing of 9736 tumors and 8587 normal samples have been deposited in the GEPIA2, retrieved from the Cancer Genome Atlas (TCGA) and the Genotype-Tissue Expression (GTEx) projects. This web-based tool (GEPIA2) is designed to provide the various expression profiles of these tumors and normal tissues or cells in a customized manner according to multiple factors such as similar gene detection, pathological stages or cancer types, and patient survivability, etc. (http://gepia2.cancer-pku.cn/#index).

The mRNA expression profile of NRP1 and ACE2 in KIRC and KIRP patients are analyzed by the UALCAN website (http://ualcan.path.uab.edu/index.html) using the TCGA dataset [15]. There was a total of 605 samples in the case of KIRC, among which 72 and 533 were from controls (normal) and cancers (primary tumor), respectively. On the other hand, 32 and 290 were from controls (normal) and cancers (the primary tumor), respectively, in the case of KIRP. The survivability rate depending on NPR1 and ACE2 expressions in KIRC and KIRP was also analyzed using the UALCAN website (http://ualcan.path.uab.edu/index.html) and the TCGA dataset [15]. UALCAN is an online portal containing clinical data of 31 cancer types and cancer OMICS data such as TCGA, MET500, and CPTAC. Various kinds of analyses include relative expression of the specific gene among normal and cancer tissues/cells according to age, sex, body weight, race tumor grade, cancer stages, and survivability rate, etc. (http://ualcan.path.uab.edu/index.html).

All the genes positively correlated with NRP1 and ACE2 in both KIPC and KIRP were downloaded from the TCGA dataset of the UALCAN and processed and viewed by FunRich software (http://funrich.org/index.html) and InteractiVenn (http://www.interactivenn.net/) [16,17]. The FunRich (Functional Enrichment) analysis tool is designed for customized handling and analysis of the datasets of various proteins and genes for graphical depiction.

A total of 7703 genes that are reported to be associated with COVID-19 were extracted from the Comparative Toxicogenomics Database (CTD) and processed (http://ctdbase.org/) [18]. CTD provides information about the interaction of genes/proteins and chemicals and the relationship of disease and genes/proteins and chemicals, thereby helping to make a possible hypothesis on the disease mechanisms. These genes are associated with COVID-19 and are either involved directly in the disease mechanism, or a therapeutic target, or have a chemical interaction. For the relationship among the genes associated with NRP1, ACE2, and COVID-19, a coexpression analysis was conducted and visualized using InteractiVenn (http://www.interactivenn.net/) [16,17]. It is an online-based tool used to analyze the dataset in the form of Venn diagrams.

## 3. Results and Discussion

**Figure 1:**
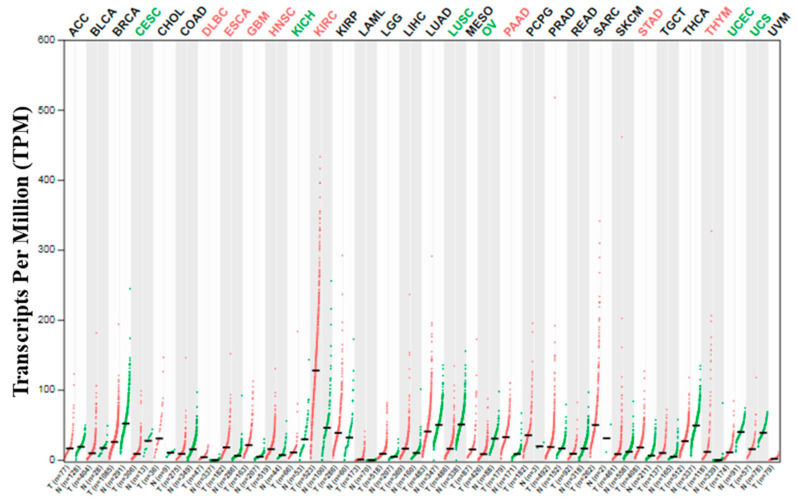
NRP1 expression profile across all tumor samples and paired normal tissues (dot plot). Each dot represents the expression of samples. T means tumor and N means normal.

KIRC and KIRP are the commonest type of kidney cancers and might cover up to 90% and 10% of the total renal cell carcinoma cases, respectively [19,20,21]. Adults, compared to children, are more susceptible to KIRC, and immune-related genes (IRGs) play a vital role in the development of this type of cancer [19]. However, both KIRC and KIRP may coexist in the same kidney [22]. Many reports showed that SARS-CoV-2 might cause direct kidney injury due to the high expression of ACE2 in the renal tissue, and multiple factors such as endothelial cell injury, host immune clearance, and immune tolerance disorders, and lipid metabolic disorders, etc. promote kidney injury [7,23,24]. Previous reports also suggested that the newly identified SARS-CoV-2 host factor NRP1 mRNA and protein expression in the kidney also control the integrity of the glomerular basement membrane [25,26]. A recent study demonstrated that high expression of NRP1 upregulates SARS-CoV-2 infections [6]. In the present study, the NRP1 expression in thirty-three (33) human cancers was checked using the GEPIA2 server [14]. Interestingly, the highest level of expression of NRP1 was detected in KIRC among all 33 human cancers (Figure 1).

**Figure 2:**
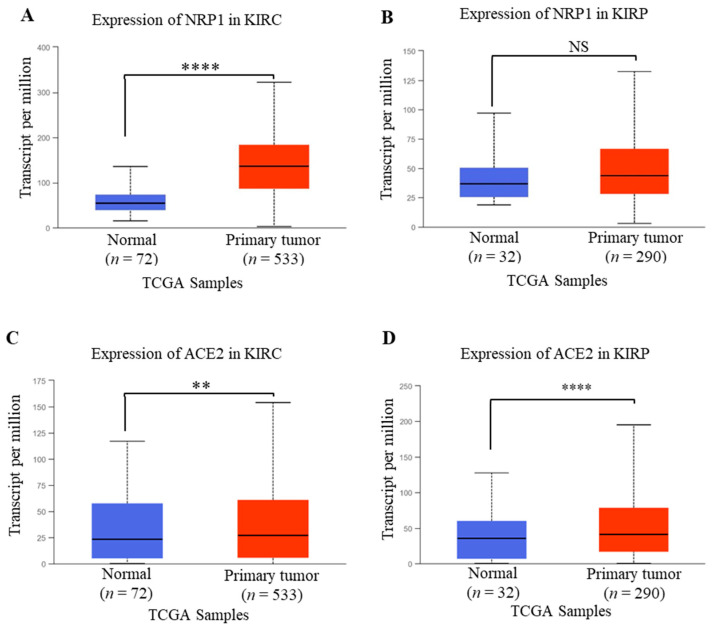
NRP1 and ACE2 mRNA expression profile in renal cancer analyzed from UALCAN. (**A**,**B**) Expression of NRP1in KIRC and KIRP. (**C**,**D**) Expression profile of ACE2 in KIRC and KIRP. The threshold *p*-values: * < 0.05, ** < 0.01, *** < 0.001, and **** < 0.0001. KIRC, KIRP, and TCGA stand for kidney renal clear cell carcinoma, kidney renal papillary cell carcinoma, and The Cancer Genome Atlas, respectively.

Though increased NRP1 expression was observed both in KIRC and KIRP compared to healthy conditions, the expression of NRP1 was found to be significantly upregulated in KIRC compared to healthy humans as measured using the TCGA dataset of UALCAN (Figure 2A,B). In addition, ACE2 expression in KIRC and KIRP was significantly upregulated compared to healthy conditions (Figure 2CD). The findings of our investigation were further supported by Tripathi et al., who analyzed the RNA-Seq data of various organs from the TCGA and GTsX databases, revealing that patients with renal carcinoma are expected to be at higher risk of SARS-CoV-2 infection [27]. Another study also revealed that renal carcinomas are reported to be associated with an increased risk of coronavirus infection due to the immune-modulating role of ACE2 in renal cancers [27]. Therefore, these results suggest that, along with the ACE2 expression, NRP1 expression might be involved in renal failure due to SARS-CoV-2 in renal cancer patients.

**Figure 3:**
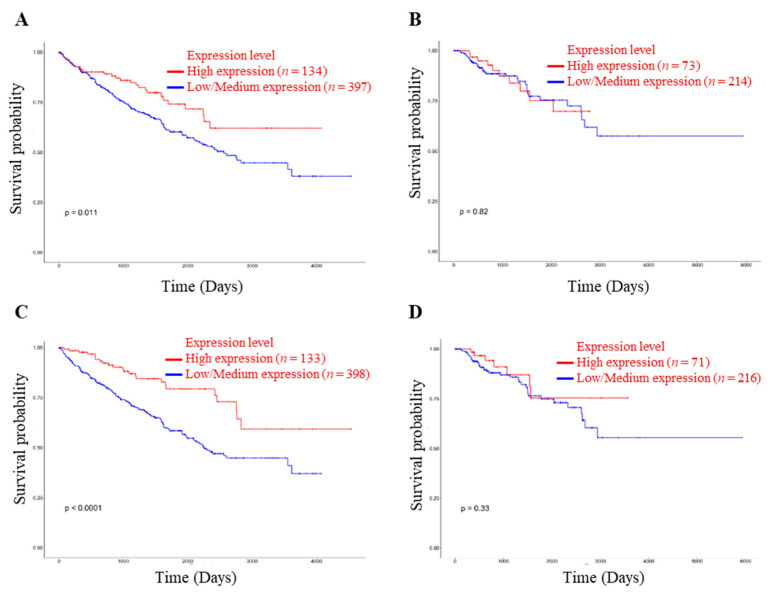
Relationship between NRP1 and ACE2 expression in renal cancer and patient’s survivability. (**A**) Effect of NRP1 expression level on KIRC patient survival. (**B**) Effect of NRP1 expression level on KIRP patient survival. (**C**) Effect of ACE2 expression level on KIRC patient survival. (**D**) Effect of ACE2 expression level on KIRP patient survival. The threshold p-value indicates the statistical significance. KIRC, KIRP, and TCGA stand for kidney renal clear cell carcinoma, kidney renal papillary cell carcinoma, and The Cancer Genome Atlas, respectively.

The survivability rate of renal cancer patients depends on the treatment and metastatic condition. In addition, renal cell carcinoma may progress in COVID-19 patients, and the mortality rate is significantly higher than in non-cancer patients [13]. Accordingly, the survivability rate is significantly correlated with expressions of NRP1 and ACE2 in KIRC patients (Figure 3A,C).

**Figure 4:**
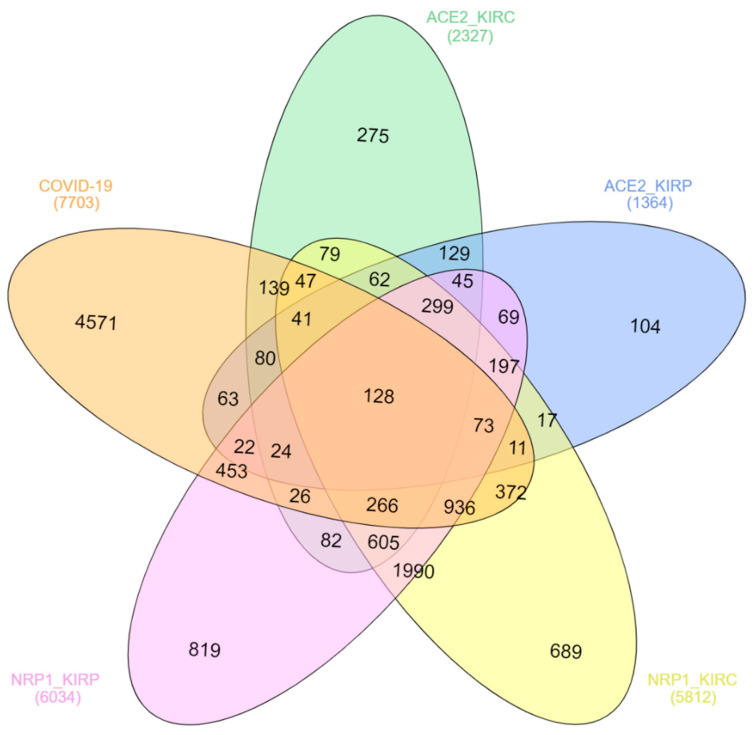
Coexpression analysis of genes involved in COVID-19, KIRC, and KIRP concerning NRP1 and ACE2 expression profiles. The indicated genes were downloaded from the databases mentioned in the Methods section, and coexpression analysis was conducted and visualized using InteractiVenn (http://www.interactivenn.net/).

Many host genes are expressed due to the SARS-CoV-2 infection, and expression variations of these genes regulate immune and other cell signaling pathways; therefore, they are involved in COVID-19 pathogenesis, disease progression, and case fatality [28]. A total of 7703 genes are reported to be associated with COVID-19 according to the Comparative Toxicogenomics Database (CTD), among which 128 genes have been overlapped with the genes positively expressed with both ACE2 and NRP1 in both KIRC and KIRP (Table 1 and Figure 4). The coexpression of many genes in COVID-19, KIRC, and KIRP concerning ACE2 and NRP1 expression might be involved in molecular mechanisms of direct kidney injury by SARS-CoV-2, cancer progression, and high mortality of renal carcinoma associated with COVID-19 patients. For example, JAK1 is one of the important pro-inflammatory cytokines that plays a role in COVID-19 pathogenesis and renal cell carcinoma (RCC), and therefore the JAK-STAT pathway is suggested as a potential target for both COVID-19 and RCC patients [29,30,31]. Tumor necrosis factor (TNF) receptor-associated factor 6 (TRAF6) is associated with signal transduction involved in kidney pathogenesis by regulating inflammation and oxidative stress [32]. TRAF6 also plays a vital role in COVID-19 pathogenesis through various cell-signaling pathways [33]. Activating transcription factor 2 (ATF2) highly expresses in RCC and regulates the transcription of CyclinB1, CyclinD1, Snail, and Vimentin and acts as a poor prognostic biomarker [34]. The expression of ATF2 is also upregulated by the SARS-CoV N protein [35].

Surprisingly, it was revealed that both the VEGF (VEGFA, VEGFC, and VEGFD) and HGF, binding proteins of NRP1, are also associated with COVID-19 [3]. Clinical studies reported that VEGF-D and HGF levels were significantly higher in the critical patients’ group, and these act as potential biomarkers of COVID-19 progression [36,37]. The elevated level of VEGF-D might be one of the important factors of blood clotting in COVID-19 patients, suggesting a therapeutic target [36,38]. Therefore, it might be suggested that a high expression of NRP1 along with ACE2 in renal carcinomas might be involved in COVID-19 pathogenesis.

## 4. Summary and Conclusions

The newly identified SARS-CoV-2 host factors, NRP1 and ACE2, are significantly upregulated in renal carcinomas. The expression of these factors is also correlated with the survivability of patients suffering from renal carcinomas. In addition, a large number of COVID-19-associated genes are coexpressed with the genes positively correlated with ACE2 and NRP1 in renal carcinomas. Therefore, along with the ACE2, a high expression of the newly identified host factor NRP1 in renal carcinomas may play a vital role in the increased risk of SARS-CoV-2 infection and high mortality of COVID-19 patients suffering from kidney cancers. The findings of this bioinformatic-based investigation will be helpful for further molecular studies and prevention and/or treatment strategies for COVID-19 patients associated with renal carcinomas.

## Figures and Tables

**Table 1:** List of 128 coexpressed genes in COVID-19, KIRC, and KIRP concerning NRP1 and ACE2 expression profiles.

Sl.	Gene	Sl.	Gene	Sl.	Gene	Sl.	Gene	Sl.	Gene
1.	ATF2	27.	ATP7B	53.	SLC35A5	79.	SLC33A1	105.	DYNLL2
2.	CYLD	28.	SLC40A1	54.	BBS10	80.	WDR31	106.	PCYOX1
3.	HBP1	29.	ALAD	55.	MED29	81.	SIK2	107.	APPL2
4.	PGK1	30.	RNF19B	56.	AP3B1	82.	TCP11L1	108.	CDC14B
5.	CFLAR	31.	RAG1	57.	TBC1D19	83.	RAB3IP	109.	DBT
6.	CHUK	32.	IFIH1	58.	KBTBD3	84.	USP8	110.	HFE
7.	RAB7A	33.	TAOK3	59.	TULP3	85.	DAZAP2	111.	MYO6
8.	SLC30A4	34.	APPL1	60.	AASDHPPT	86.	DDX18	112.	AKTIP
9.	ACOX1	35.	TGOLN2	61.	TGFBRAP1	87.	PEX12	113.	AFF4
10.	TEAD1	36.	CANX	62.	CRIPT	88.	DUSP11	114.	RIOK3
11.	MTMR6	37.	WIPF2	63.	TM9SF2	89.	SBNO1	115.	RRAGC
12.	PPP2R1B	38.	STOM	64.	RAB21	90.	VPS33A	116.	RNF13
13.	RAB1A	39.	PNMA2	65.	MAGT1	91.	FBXO3	117.	IDE
14.	NCOA4	40.	TSR1	66.	ANKRD27	92.	SAP30L	118.	PAFAH1B1
15.	AP1G1	41.	MAP3K13	67.	DHX29	93.	MTRR	119.	ENDOD1
16.	JAK1	42.	PIGN	68.	DENND5B	94.	RANBP2	120.	CLTC
17.	ATF7IP	43.	APBB1	69.	TMLHE	95.	EPB41L4A	121.	PHLDB2
18.	MIA2	44.	PSMD11	70.	BIRC2	96.	NLRX1	122.	FZD1
19.	TSHZ1	45.	VPS24	71.	DCTN1	97.	SGCB	123.	CREB5
20.	SPAG9	46.	TBL1X	72.	NARS2	98.	ZFP91	124.	ADD3
21.	MLEC	47.	AP2B1	73.	SHPK	99.	FARS2	125.	SLC1A1
22.	TRAF6	48.	INVS	74.	TTC26	100.	PURA	126.	PPP2CA
23.	IFIT1	49.	NCOA1	75.	SLU7	101.	FRMD4B	127.	APC
24.	RPS6KA3	50.	PIK3R4	76.	TXNDC9	102.	MDM1	128.	NCOR1
25.	FTO	51.	TMEM127	77.	CHIC1	103.	PCBP1		
26.	CLIC4	52.	TTC21B	78.	ZSWIM5	104.	BICC1		

## Data Availability

All the data and materials used in this study have been mentioned in the article.
